# Stroke in Young Adults: A Narrative Review of Clinico-Radiological Approach to Twenty Key Etiologies

**DOI:** 10.3390/clinpract16100180

**Published:** 2026-09-29

**Authors:** Bruno Kusznir Vitturi, Felipe Torres Pacheco, Rubens José Gagliardi

**Affiliations:** 1 Division of Neurology, University of Milan, 20133 Milan, Italy; 2 Division of Neuroradiology, Faculty of Medical Sciences of Santa Casa de São Paulo, São Paulo 01225-010, Brazil; 3 Division of Neurology, Faculty of Medical Sciences of Santa Casa de São Paulo, São Paulo 01225-010, Brazil

**Keywords:** stroke, cerebrovascular diseases, stroke etiology, neuroimaging, young adults

## Abstract

**Background:** Stroke in young adults is increasing worldwide and is associated with substantial long-term disability. Unlike stroke in older populations, young patients frequently present with uncommon etiologies that require specific diagnostic approaches and targeted secondary prevention strategies. Early recognition of these causes remains a major clinical challenge. **Objective:** This study aimed to provide a comprehensive review of the principal uncommon causes of stroke in young adults, emphasizing their clinical characteristics and neuroimaging findings relevant to etiological diagnosis. **Methods:** A narrative review was conducted focusing on cerebrovascular disorders that disproportionately affect young adults, including arterial dissections, patent foramen ovale, moyamoya disease, reversible cerebral vasoconstriction syndrome, cerebral venous thrombosis, thrombophilias, autoimmune diseases, infections, genetic disorders, malignancy, and cardiac conditions. Particular attention was given to neuroradiological patterns and imaging modalities useful for diagnosis. **Results:** Numerous uncommon etiologies contribute significantly to stroke in young adults and often occur in the absence of traditional vascular risk factors. Neuroimaging plays a pivotal role in diagnosis, helping identify characteristic findings such as double-lumen signs in arterial dissections, collateral vessel networks in moyamoya disease, reversible multifocal vasoconstriction in RCVS, venous filling defects in cerebral venous thrombosis, and distinctive imaging signatures associated with genetic, infectious, inflammatory, and neoplastic disorders. Recognition of these patterns can substantially improve diagnostic accuracy and guide appropriate secondary prevention. **Conclusions:** Stroke in young adults encompasses a broad spectrum of etiologies that differ considerably from those observed in older populations. Awareness of characteristic clinical and neuroimaging features is essential for accurate etiological investigation and implementation of effective preventive strategies.

## 1. Introduction

**Figure 1:**
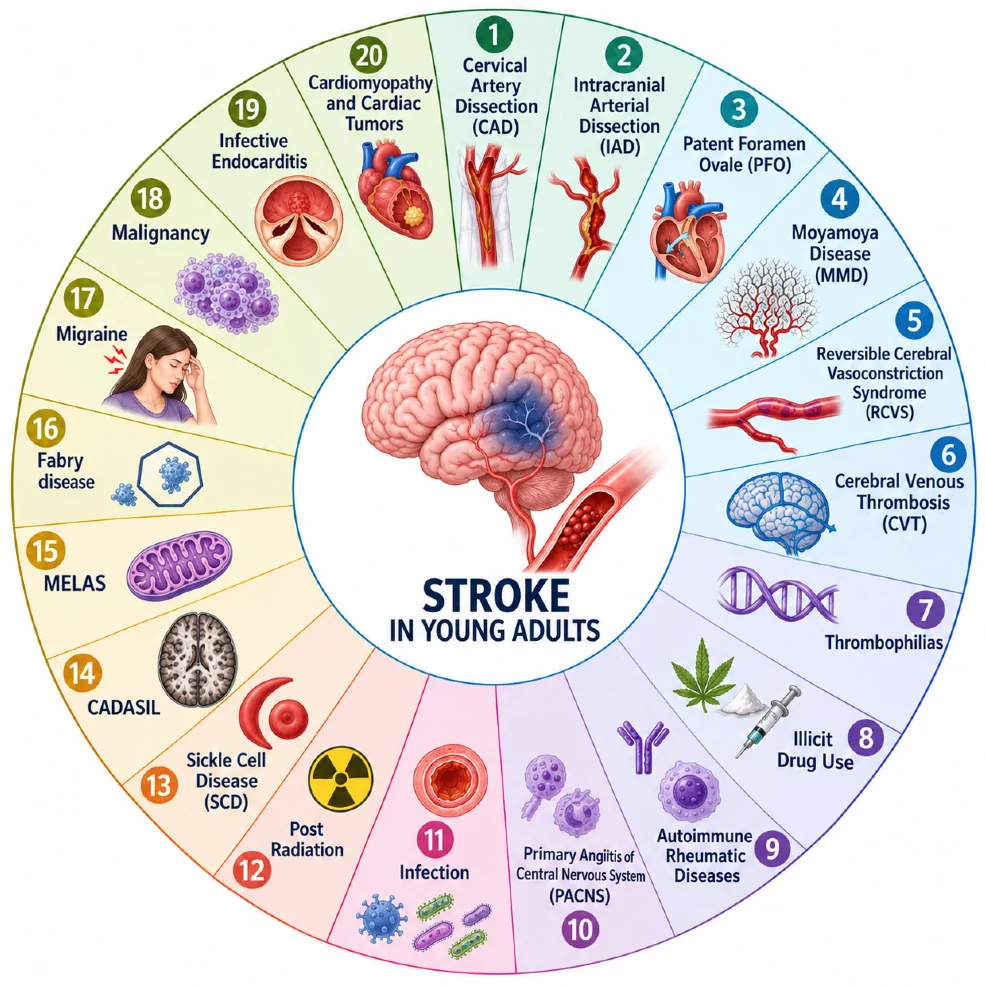
Overview of the main causes of stroke in young adults.

The incidence of stroke in young adults (<50 years) has increased significantly in recent years [1]. At the same time, the rate of recurrent stroke among young adults has remained stable over the last decade, highlighting the need for a better understanding of the most common etiologies and for improvement of secondary prevention strategies in this population [2]. These conditions typically manifest in young adults in the absence of traditional cerebrovascular risk factors. Indeed, although the classical causes of stroke remain relevant, uncommon etiologies are proportionally more frequent in younger patients. The main causes will be presented separately (Figure 1).

There is considerable variation in the cutoff used to define a “young adult.” We adopted <50 years as the definition because it is the most frequently used and widely accepted threshold in the literature. To improve the transparency of the literature identification process, we performed a search of the PubMed database using combinations of the following search terms: “stroke,” “ischemic,” “brain infarction,” “cerebral infarction,” “thrombosis,” “young,” “neuroimaging,” “neuroradiolog,” “diagnosis,” “early-onset,” and “juvenile.”* Original articles, case reports, and case series were considered eligible. No restrictions were applied regarding publication date or language. Based on this literature search and our clinical experience, we selected the 20 etiologies considered most relevant and frequently encountered in clinical practice for inclusion in this narrative review. Among these etiologies, some are considerably more common than others. Cervical artery dissection and PFO-associated stroke, for example, are substantially more frequent than rare conditions such as MELAS or CADASIL.

## 2. Cervical Artery Dissection (CAD)

**Figure 2:**
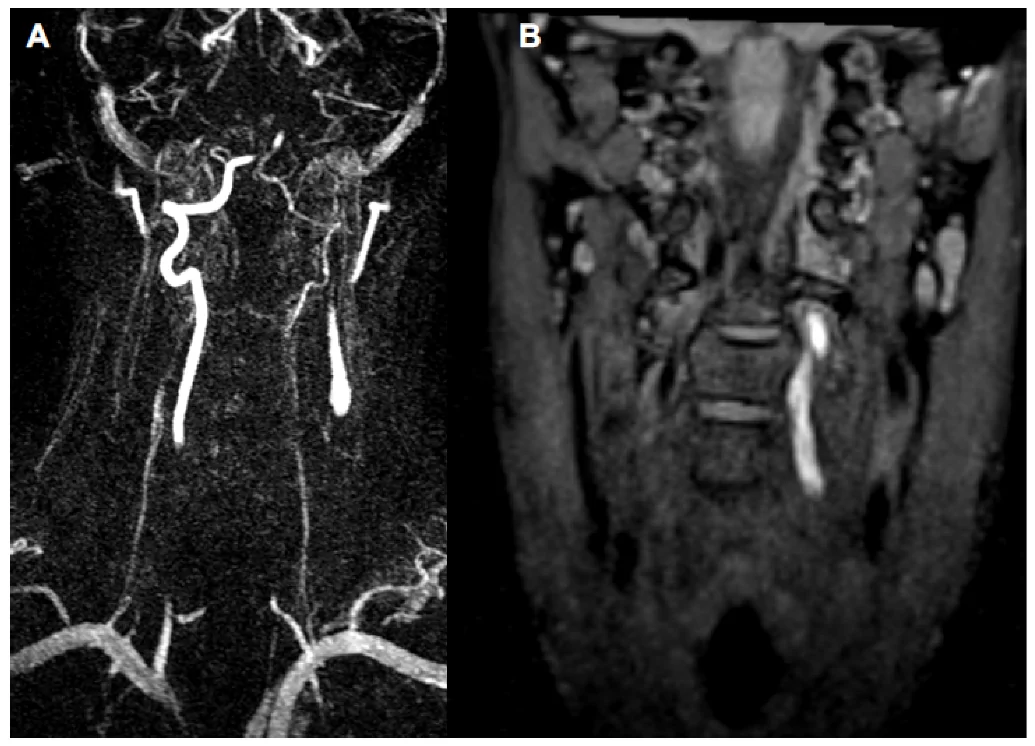
Cervical MR angiography and vessel wall MRI in left vertebral artery dissection. (**A**) Cervical MR angiography demonstrating diffuse hypoplasia of the left vertebral artery with near-occlusion (subocclusion) at its middle third. (**B**) Vessel wall MRI of the cervical segment revealing a hyperacute mural thrombus at its middle third causing near-occlusion of the left vertebral artery.

CAD is a multifactorial disease that may occur spontaneously or in association with trauma. Minor traumatic events, including motor vehicle accidents, sporting activities, whiplash injuries, severe coughing, and cervical manipulations, represent the most common causes of CAD in the young population [3,4]. Additional risk factors include infection, migraine, inherited connective tissue disorders, hyperhomocysteinemia, and the 677TT genotype of the 5,10-methylenetetrahydrofolate reductase gene (MTHFR 677TT) [5]. The classical clinical presentation consists of neurological deficits related to cerebral ischemia associated with local symptoms resulting from direct injury to the cervical arterial wall, most commonly Horner syndrome, neck pain, and unusual headache, and, less frequently, lower cranial nerve palsies [6]. Neuroimaging plays a central role in confirming the diagnosis, with characteristic findings identifiable on magnetic resonance angiography (MRA), computed tomography angiography (CTA), or conventional angiography. The most typical and specific imaging feature is an enlarged artery with a double lumen on axial sections, with an intraluminal thrombus in the false lumen. This finding can be demonstrated particularly well on T1-weighted fat-saturated magnetic resonance imaging (MRI) sequences (Figure 2). Another highly characteristic feature is the presence of gradually tapering arterial stenosis or occlusion [7]. Other possible radiological findings include dissecting aneurysm formation and the presence of an intimal flap [8]. The most frequent sites of extracranial dissection include the distal cervical internal carotid artery (ICA) from the common carotid artery and the V2–V3 segments of the vertebral arteries. Intracranial arterial dissections demonstrate a more variable anatomical distribution [3].

## 3. Intracranial Arterial Dissection (IAD)

The mechanisms underlying the pathogenesis of IAD are less well understood compared with CAD. Fundamentally, IAD is associated with the particular histological characteristics of intracranial vessels. Intracranial arteries lack an external elastic lamina, possess a thicker internal elastic lamina, contain fewer elastic fibers within the tunica media, and have a thinner adventitia when compared with extracranial arteries [9]. From a clinical perspective, IAD also presents with symptoms related to cerebral ischemia; however, unlike CAD, it is more commonly associated with subarachnoid hemorrhage and with mass effect involving the brainstem or cranial nerves [10,11]. Radiological diagnosis is often more challenging, partly because of lower clinical suspicion due to the predominance of nonspecific ischemic symptoms. Imaging findings suggestive of IAD include the presence of the pearl-and-string sign (alternating widening and narrowing of the lumen), contrast stagnation within the affected arterial segment, or irregular vessel stenosis [9]. Irregular stenosis or occlusion may also be observed, as well as an aneurysm, most commonly fusiform. The use of cone-beam CT angiography may be particularly useful in patients with suspected IAD [12].

## 4. Patent Foramen Ovale (PFO)

PFO is an important cause of embolic stroke of undetermined source (ESUS) [13]. PFO is present in approximately 25% of adults but is responsible for only about 10% of stroke cases in young individuals. Establishing with certainty whether a PFO is causally related to stroke is essential for determining the most appropriate therapeutic management. The RoPE (Risk of Paradoxical Embolism) score uses different clinical characteristics—such as age, history of stroke or transient ischemic attack, presence of diabetes, arterial hypertension, smoking status, and evidence of cortical infarction on imaging studies—to estimate the likelihood that an embolic stroke of undetermined source is related to the presence of a PFO. The PASCAL classification is the subsequent step that helps determine the probability of a causal association [14,15].

On neuroimaging, PFO-associated stroke frequently presents with vertebrobasilar territory ischemia or multisite brain involvement in young patients. The vertebrobasilar circulation is more frequently affected in PFO-related stroke, possibly because it receives less adrenergic innervation and consequently exhibits a greater increase in blood flow during the Valsalva maneuver compared with the anterior circulation [16]. Large infarcts, superficially located lesions, and the absence of previous infarcts on neuroimaging are associated with a higher likelihood of PFO-related stroke [17]. Patients with larger right-to-left shunts (RLSs) more frequently demonstrate posterior circulation infarcts, as well as small or multiple cortical lesions [18].

## 5. Moyamoya Disease (MMD)

MMD is a chronic, non-inflammatory, non-atherosclerotic cerebrovascular disorder characterized by progressive stenosis of the terminal portion of the internal carotid artery and its proximal branches, associated with the development of an abnormal collateral vascular network [19]. The disease predominantly affects individuals of East Asian ancestry and represents an important cause of stroke in both young adults and children. In pediatric populations, the disease is often underdiagnosed and may present with transient ischemic attacks as well as overt and covert ischemic lesions, which are associated with an increased risk of intellectual impairment. In adults, MMD more commonly manifests with intracranial hemorrhage, although ischemic stroke also remains highly prevalent [19,20]. The term “moyamoya syndrome” (MMS) refers to moyamoya disease-like vascular changes occurring in association with underlying congenital or acquired conditions.

**Figure 3:**
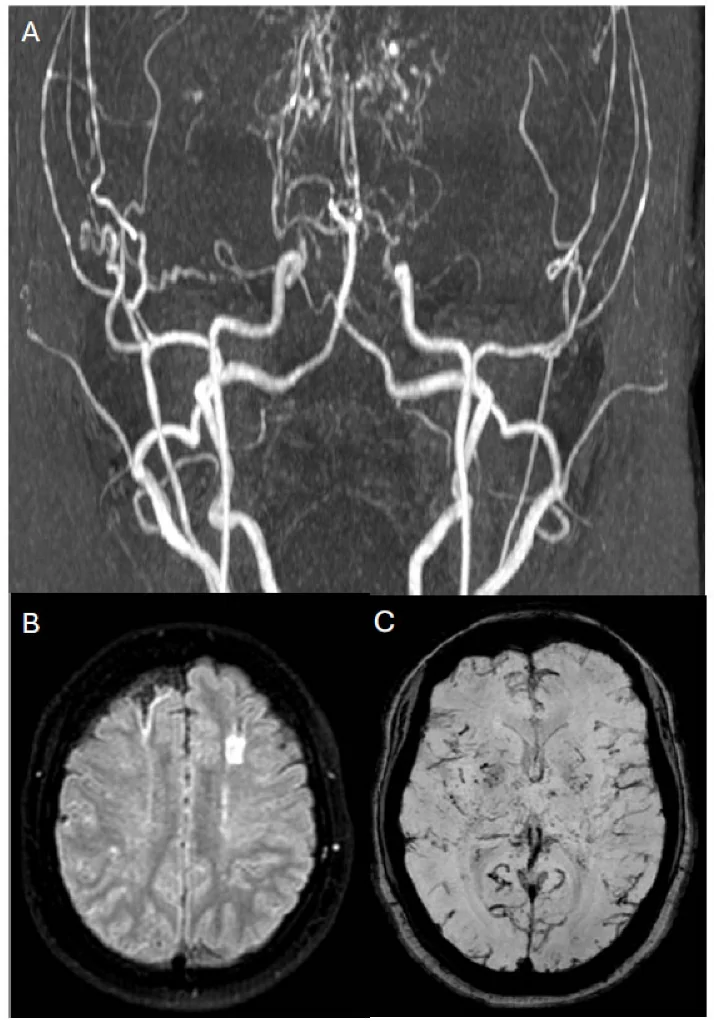
Intracranial MR angiography, FLAIR, and susceptibility-weighted imaging in moyamoya disease. (**A**) Intracranial MR angiography demonstrating occlusion of the terminal bifurcation of both internal carotid arteries, involving the proximal segments of the anterior and middle cerebral arteries bilaterally, with development of a Moyamoya collateral pattern in the centroencephalic regions. (**B**) Axial FLAIR image showing sequelae of watershed infarcts in both cerebral hemispheres. (**C**) Axial susceptibility-weighted imaging (SWI) demonstrating neocollateralization vessels in the centroencephalic regions, with tortuous and ectatic vessels in the bilateral nucleocapsular regions.

From a radiological perspective, the disease characteristically leads to the formation of collateral vessels with the angiographic appearance of a “puff of smoke” (Figure 3). Indeed, the definitive diagnosis of MMD relies on conventional cerebral angiography, which also enables classification of the disease into six stages as proposed by Suzuki et al. [21]. CT angiography or MR angiography may be useful for identifying steno-occlusive vascular changes and abnormal collateral networks that are disproportionate to the patient’s age [22]. High-resolution vessel wall MRI may additionally demonstrate concentric narrowing of the vessel wall [19].

## 6. Reversible Cerebral Vasoconstriction Syndrome (RCVS)

RCVS is clinically characterized by recurrent thunderclap headaches and diffuse vasoconstriction of cerebral arteries that typically resolves spontaneously within 3 months. The condition is more common in women. The hallmark clinical feature is the occurrence of a severe headache of maximal intensity, frequently described as explosive, with abrupt onset and peak intensity reached within 1 min [23,24].

Although the syndrome may follow a monophasic course, recurrence of headache within up to 4 weeks is observed in most patients. Headache is frequently associated with transient or permanent focal neurological deficits and seizures. RCVS is associated with several well-established triggers described in the literature, including stressful situations, coughing, showering, swimming, laughing, sexual activity, strenuous physical exercise, exposure to licit or illicit vasoactive substances, immunosuppressive drugs, immunomodulatory agents, antidepressants, endovascular procedures, the puerperium, CAD, catecholamine-secreting tumors, and hypercalcemia [24].

Neurological deficits are usually transient, predominantly visual, and may be mistaken for migraine aura. Neurological examination is often normal; when persistent focal deficits are present, ischemic or hemorrhagic complications should be suspected [23]. Although RCVS presents abruptly and dramatically, the prognosis is generally favorable, and the disease is typically self-limited.

The initial radiological evaluation should include non-contrast brain CT to exclude the main differential diagnosis, subarachnoid hemorrhage (SAH). If CT findings are normal, lumbar puncture should be performed for definitive exclusion of SAH. Neuroradiological studies may initially be normal or may already demonstrate the three principal complications of the syndrome: convexity subarachnoid hemorrhage, intraparenchymal hemorrhage, and cerebral ischemia.

**Figure 4:**
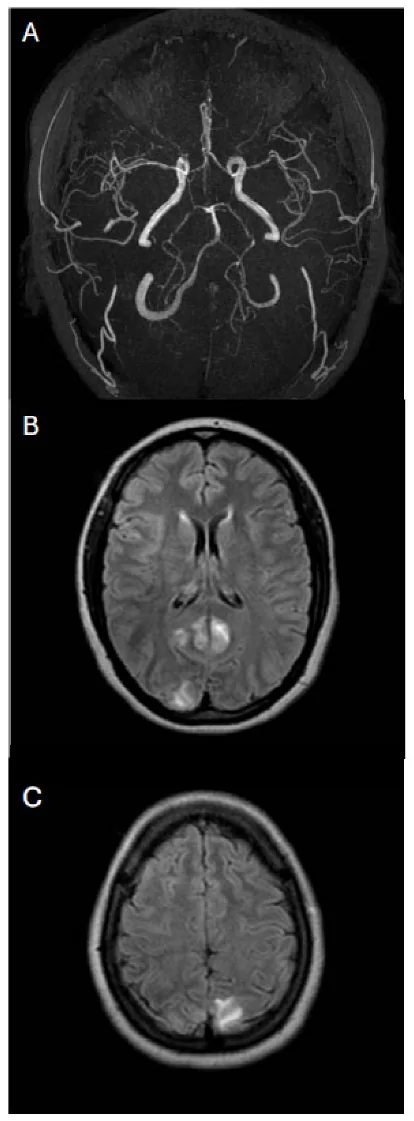
Intracranial MR angiography (TOF) and FLAIR imaging in reversible cerebral vasoconstriction syndrome (RCVS). (**A**) Time-of-flight (TOF) intracranial MR angiography demonstrating a vasoconstriction pattern involving the anterior and middle cerebral arteries, and more prominently the posterior cerebral arteries, with alternating focal ectasias and constrictions. (**B**,**C**) Axial FLAIR images showing vasogenic edema involving the subcortical white matter of the occipital and posterior parietal regions. Follow-up imaging demonstrates complete resolution of both the angiographic abnormalities and the parenchymal changes.

The diagnosis is primarily based on the clinical presentation but depends on angiographic evaluation, with digital subtraction angiography (DSA) remaining the gold standard. Angiographic studies typically demonstrate segmental arterial narrowing and dilatation involving one or more vessels, with regression of these abnormalities on follow-up imaging days after symptom onset [24]. The disease predominantly affects medium- to large-caliber vessels. Peak vasoconstriction usually occurs during the second week after symptom onset [25]. A definitive diagnosis of RCVS requires demonstration of reversibility of the vasoconstriction within 12 weeks of onset [24] (Figure 4). Vessel wall imaging may be particularly useful in differentiating RCVS from cerebral vasculitis [25].

## 7. Cerebral Venous Thrombosis (CVT)

The clinical presentation of CVT is highly variable, which frequently leads to underdiagnosis. In general, symptoms result from increased intracranial pressure, focal parenchymal injury, or encephalopathy. Symptom onset is typically insidious and subacute. Headache is the most common symptom. Additional manifestations include nausea, visual disturbances, papilledema, diplopia, cranial nerve palsies, altered mental status, seizures, and focal neurological deficits [26,27].

CVT should be particularly suspected in patients presenting with these symptoms in the presence of known risk factors. Important risk factors include oral contraceptive use, hormonal therapy, pregnancy, puerperium, dehydration, obesity, hematological disorders, inflammatory diseases, and infectious conditions [27,28].

CT or MRI may demonstrate hyperdensity within the affected venous structures or reveal imaging findings related to venous obstruction at the tissue level, including venous infarction, juxtacortical hemorrhage, intracranial hypertension, and hydrocephalus [26,28]. Findings that may already be visible on non-contrast CT include the cord sign, defined as a serpiginous or linear hyperdensity within a cortical vein, and the dense triangle sign.

**Figure 5:**
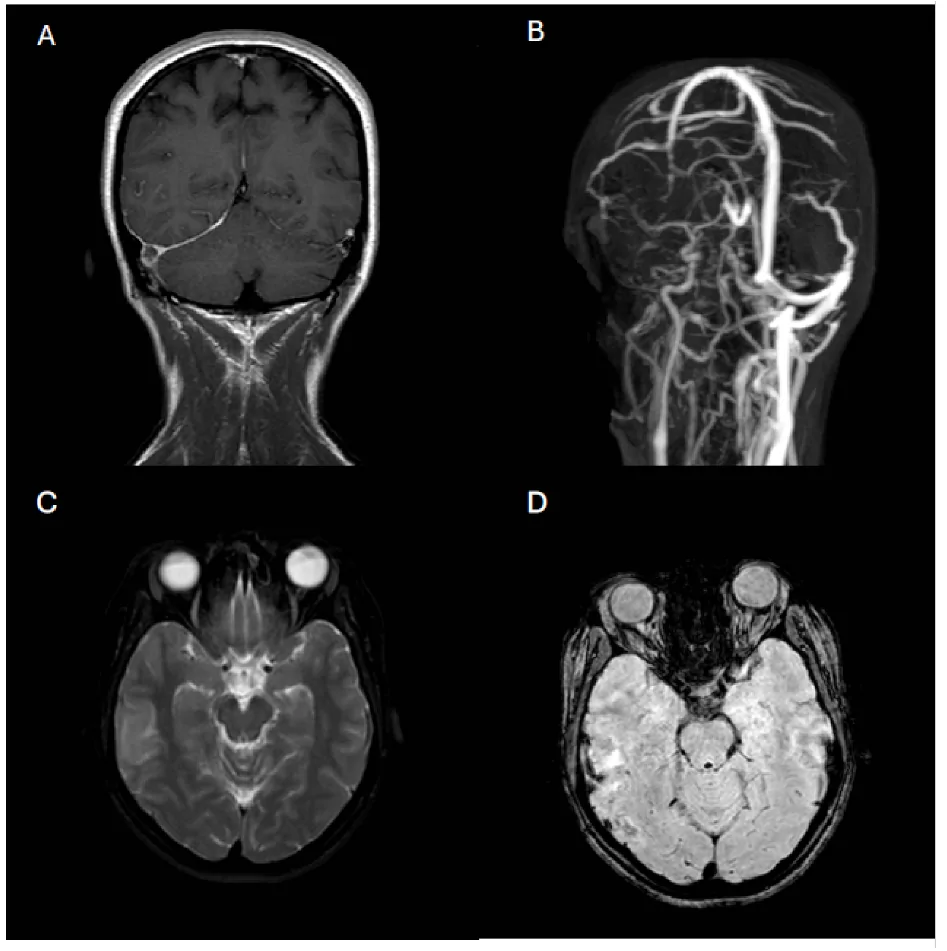
MRI and MR venography in cerebral venous sinus thrombosis. (**A**) Coronal post-contrast image demonstrating a thrombus in the right transverse sinus. (**B**) MR venography showing absence of opacification of the lateral venous sinuses and right jugular vein, confirming the diagnosis of venous sinus thrombosis. (**C**) Parenchymal signal changes in the venous drainage territory of the vein of Labbé. (**D**) Linear susceptibility artifact on susceptibility-weighted imaging (SWI) indicating thrombosis of the right cortical vein of Labbé.

When CVT is clinically suspected, CT venography or MR venography should be performed, as these modalities may demonstrate venous filling defects (Figure 5). MR venography offers the additional advantage of avoiding contrast administration through the use of time-of-flight (TOF) techniques [26].

## 8. Thrombophilias

Thrombophilias are a rare cause of stroke; however, they become particularly relevant in cases of stroke affecting young individuals. Thrombophilic disorders may be inherited or acquired, with the most commonly identified conditions including factor V Leiden mutation, the G20210A prothrombin gene mutation, protein C and protein S deficiency, antithrombin III deficiency, and antiphospholipid syndrome [5].

The role of thrombophilia screening remains controversial; however, stroke occurring at a young age, cryptogenic stroke classification, and stroke occurring during states of high estrogen exposure increase the pretest probability of an underlying thrombophilic disorder [29]. Investigation of previous thromboembolic events in other vascular territories, recurrent spontaneous miscarriages, and a family history of thromboembolic disease, particularly at an early age, is essential.

There are no highly specific neuroimaging patterns for the diagnosis of thrombophilia-related stroke. Nevertheless, the presence of multiple acute ischemic lesions involving multiple vascular territories, with a tendency toward lesion confluence and less frequent involvement of the anterior circulation, associated with silent infarcts, may increase the likelihood of this etiology, particularly in patients with negative investigations for more common stroke mechanisms [30].

## 9. Illicit Drug Use

Several forms of substance misuse may result in ischemic or hemorrhagic stroke. Abuse of cannabis, cocaine, and amphetamines represents a well-recognized cause of stroke in young individuals. Among these substances, amphetamines and cocaine appear to be more strongly associated with stroke occurrence [31].

The mechanisms underlying stroke in individuals with substance dependence are multifactorial and vary according to the drug involved. In general, illicit drugs may induce direct vascular wall injury, increase blood pressure, promote vasoconstriction, facilitate aneurysm rupture, and increase the risk of cardiac arrhythmias [32]. Cannabis use disorder has been associated with an increased risk of overall stroke and large-artery stroke, whereas cocaine dependence appears to be more strongly associated with cardioembolic stroke and intracerebral hemorrhage [31,33].

More recently, the substantial increase in the variety of available illicit substances has complicated the understanding of stroke pathophysiology in these patients. It should also be emphasized that illicit drug use is frequently not directly acknowledged by patients, which may contribute to difficulties in etiological determination and to underestimation of the magnitude of the problem.

There are no pathognomonic radiological findings associated with stroke related to illicit substance abuse. However, in cases of strong clinical suspicion, neuroimaging may support the initial diagnostic hypothesis, as patients with substance dependence may already demonstrate chronic imaging abnormalities, including multiple silent infarcts involving different vascular territories, severe vascular leukoencephalopathy, and diffuse or focal cortical atrophy disproportionate to the patient’s age [34].

## 10. Autoimmune Rheumatic Diseases

Autoimmune rheumatic diseases may eventually cause ischemic stroke, intraparenchymal hemorrhage, and cerebral venous thrombosis in young individuals. Systemic lupus erythematosus and systemic vasculitides confer particularly high cerebrovascular risk, although virtually all autoimmune rheumatic diseases have been implicated as risk factors for stroke in the young population [35,36]. Among systemic vasculitides, Behçet disease and ANCA-associated vasculitides are among the most important causes of stroke [37,38].

The underlying pathophysiological mechanisms are generally related to inflammatory involvement of small- and medium-sized cerebral vessels, predisposing patients to cerebral ischemia. In addition, these patients have an increased risk of subclinical atherosclerosis [39]. Furthermore, chronic systemic inflammation itself is recognized as an important independent risk factor for stroke [40].

Neuroimaging findings are not specific in rheumatic disease-related stroke. The most frequently observed parenchymal abnormality is cerebral small vessel disease. Additionally, imaging may reveal evidence of central nervous system inflammation occurring concomitantly with radiological signs of cerebrovascular injury.

## 11. Primary Angiitis of the Central Nervous System (PACNS)

PACNS is a rare vasculitic disorder of unknown etiology involving the arteries of the brain, spinal cord, and leptomeninges in the absence of systemic vasculitis [41]. There is no predilection for a specific cerebral vessel, and the disease more commonly affects male individuals.

PACNS typically presents with an insidious onset and rarely manifests as an isolated acute stroke. The interval between symptom onset and diagnosis may extend up to 6 months. Other common manifestations include headache, psychiatric disturbances, cognitive decline, and seizures [42].

Typical neuroradiological findings include alternating areas of smooth-walled segmental narrowing and dilatation of cerebral arteries, arterial occlusions involving multiple cerebral vessels, and the absence of significant proximal atherosclerosis. Digital subtraction angiography (DSA) remains the gold standard for diagnosis, and normal MR angiography findings do not exclude the disease [41].

High-resolution vessel wall MRI may demonstrate vessel wall hyperintensity, supporting the diagnosis. Additional MRI findings may include gadolinium enhancement of the brain parenchyma or meninges [41,43]. Infarcts are generally bilateral, involve multiple vascular territories, and may affect both cortical and subcortical regions. Infarcts of different ages may also be identified [42,43].

## 12. Infection

Several central nervous system (CNS) infections may cause stroke. The underlying mechanisms are diverse and include multifocal vasculopathy, focal infiltrative vasculitis, direct vessel wall invasion, and thrombus formation [44].

During the diagnostic evaluation, it is essential to determine whether the patient is immunocompromised, as some CNS infections are exceedingly rare in immunocompetent individuals. It should also be emphasized that neuroinfectious diseases may act as stroke mimics or may cause silent cerebral infarctions, further highlighting the complexity of stroke diagnosis in these patients.

Additionally, certain infections, such as varicella zoster virus (VZV) and Chlamydia pneumoniae, substantially increase the risk of stroke during the years following apparent clinical resolution of the infection [45,46]. Infection-associated stroke does not necessarily result from direct CNS infection but may also occur secondary to systemic inflammatory and prothrombotic states associated with generalized infections. Indeed, pneumonia is the most common cause of post-stroke infection [47].

Neuroradiological findings are highly variable depending on the underlying CNS infection. Virtually any neuroinfectious disease may be associated with cerebrovascular lesions. Patients with VZV-associated vasculitis commonly demonstrate ischemic lesions within the middle cerebral artery (MCA) or anterior cerebral artery (ACA) territories, classically ipsilateral to the cutaneous manifestations of the disease when present [44]. Basal ganglia infarcts are particularly frequent, typically occurring at the gray–white matter junction. Some infarcted areas may demonstrate contrast enhancement. Vessel wall imaging and angiography may be extremely useful in identifying the associated vasculitic process. Involvement of the central retinal artery is also common in VZV infection [48].

Herpes simplex virus (HSV-1 and HSV-2) is a common cause of meningoencephalitis that may evolve with cerebrovascular complications. HSV-1 is more frequently associated with hemorrhage, whereas HSV-2 is more commonly associated with ischemic stroke. In these cases, in addition to the classic radiological findings of viral encephalitis, angiography may demonstrate cerebral vasculitis predominantly involving large-caliber vessels [49].

Cerebrovascular complications are common in patients with tuberculous meningitis (TBM), with lacunar ischemic stroke representing the most frequent complication. Most ischemic lesions are concentrated within the so-called “tubercular zone” or “ischemic zone.” The tubercular zone includes the head of the caudate nucleus, the anteromedial thalamus, and the anterior limb and genu of the internal capsule. The ischemic zone comprises the posterior limb of the internal capsule, the lentiform nucleus, and the posterolateral thalamus [44].

Contrast-enhanced MRI is essential for identifying meningeal enhancement at the base of the brain, a characteristic finding of TBM that is associated with increased stroke risk [50]. The classic angiographic triad of TBM includes segmental narrowing of the supraclinoid portion of the internal carotid artery, wide sweeping of the pericallosal arteries, and delayed circulation within the middle cerebral artery associated with scant collateral circulation and early draining veins. A moyamoya-like angiographic pattern may also be observed [51]. It should be emphasized, however, that angiography may remain normal in some patients with TBM-associated stroke.

Radiological findings in neurosyphilis-related stroke are relatively nonspecific. Angiography is essential for the diagnosis of the meningovascular form of the disease, although angiographic findings overlap with those of other infectious vasculitides. In meningovascular neurosyphilis, gadolinium-enhanced MRI may demonstrate meningeal enhancement. In the gumma form, ischemic lesions may also occur, while MRI typically reveals nodular or mass-like lesions that are isointense on T1-weighted imaging and slightly hyperintense on T2/FLAIR sequences, associated with extensive surrounding edema [52].

In CNS aspergillosis, cortical or subcortical strokes may occur with or without hemorrhagic transformation. Cerebral angiography may demonstrate findings compatible with fungal vasculitis and may identify fungal mycotic aneurysms. Fungal mycotic aneurysms differ from bacterial aneurysms in that they are more frequently fusiform, may involve longer vascular segments, and tend to affect proximal intracranial arteries [53].

Direct neuroinfectious lesions caused by aspergillosis may appear as multiple T2-hypointense lesions with irregular ring-like contrast enhancement, most commonly located within the frontal lobes or cerebellopontine angle, with or without associated dural enhancement. Additional imaging findings suggestive of fungal etiology include adjacent osseous abnormalities, such as sclerotic thickening, bone erosion, and remodeling near the paranasal sinuses or middle ear, depending on the route of infection entry [53,54].

Neurocysticercosis represents a rare but well-recognized etiology of cerebrovascular disease, most commonly manifesting as ischemic stroke. Brain MRI typically reveals multiple cortical and subcortical infarctions in association with subarachnoid or cisternal cystic lesions. Vascular imaging may demonstrate diffuse inflammatory vasculopathy affecting both the carotid and vertebrobasilar arterial territories. Cerebrospinal fluid analysis frequently discloses lymphomonocytic pleocytosis, while immunological assays, such as ELISA, support the diagnosis by detecting antibodies against Taenia solium [55].

## 13. Post-Radiation

Exposure of the CNS to radiation, particularly radiotherapy, is a recognized cause of stroke in young individuals. The risk does not depend on the neoplasm itself but rather on the amount of radiation received [56]. Radiation causes direct damage to the arterial wall, leading to vessel wall thickening, thrombosis, luminal occlusion, and occasional telangiectasias [57]. More specifically, radiation induces direct endothelial damage associated with substantial microvascular impairment [58]. Stroke-related symptoms may begin as early as a few days after the radiotherapy session.

Neuroimaging may reveal radiation-induced cerebral contrast enhancements (RICEs), which are radiological alterations frequently observed after photon and proton therapy and are associated with an increased cerebrovascular risk [59]. In the acute phase, there are no highly specific findings strongly suggestive of stroke etiology. The key element is recognizing an infarcted area that is compatible with the field of radiotherapy exposure. Small vessel disease, microbleeds, and carotid artery stenosis are more prevalent among patients who have received radiation therapy [60,61,62]. Radiation involving the cervical spine may particularly cause distal internal carotid artery narrowing, with or without the development of collateral vessels [5].

## 14. Sickle Cell Disease (SCD)

Stroke is a potentially devastating and fatal complication of SCD. Patients with SCD are at increased risk of stroke due to multiple disease-related mechanisms, including vasculopathy, endothelial injury, hypercoagulability, and hemodynamic instability [61]. The prevalence of stroke among patients with SCD is approximately 5% [63,64]. Stroke may occur in both HbSS and HbSC phenotypes and in both adults and children [65]. Up to 40% of patients have silent ischemic lesions, which help explain the cumulative functional and cognitive impairment observed in individuals with SCD over the years [66,67].

A key aspect and particularity of stroke prevention in this group of patients is the systematic screening for cerebral vasculopathy using transcranial Doppler ultrasonography, which can identify more vulnerable individuals and support treatment intensification [68]. Randomized and prospective studies demonstrated that chronic transfusion therapy in children with abnormal transcranial Doppler findings dramatically reduced the risk of stroke compared with standard care [69,70]. Hydroxyurea may also represent an effective strategy for stroke prevention in these individuals [71].

## 15. CADASIL

CADASIL is a generalized small vessel disease caused by mutations in the NOTCH3 gene located on chromosome 19, which plays a fundamental role in cerebral vessel maturation during the perinatal period [72]. The pathophysiology involves loss of smooth muscle cells from the tunica media of small cerebral arterioles, associated with deposition of granular osmiophilic material. CADASIL is a genetic disorder characterized by autosomal dominant arteriopathy with subcortical infarcts and leukoencephalopathy. The disease follows a progressive course.

CADASIL is classically characterized by a tetrad consisting of migraine with aura, with an average onset around 30 years of age; recurrent subcortical ischemic strokes, typically occurring between 40 and 59 years of age; mood and psychiatric disorders; and cognitive impairment, usually beginning around the age of 50 years [73,74].

The typical presentation is that of a young patient with recurrent strokes and a history of migraine who progressively develops cognitive decline leading to vascular dementia. Not uncommonly, the diagnosis is established during the third decade of life in patients presenting with migraine with aura and an unexpected leukoencephalopathy on MRI. From this age onward, ischemic stroke recurrences become more frequent, and neuropsychiatric manifestations gradually emerge and progress toward dementia.

MRI demonstrates hypointense lesions on T1-weighted images and hyperintense lesions on T2/FLAIR sequences, affecting the subcortical white matter in a confluent pattern, with preferential involvement of the anterior temporal lobes and the external capsule [75]. Brain atrophy is a strong predictor of dementia in these patients [76].

## 16. MELAS

The acronym refers to a syndrome characterized by mitochondrial myopathy, encephalopathy, lactic acidosis, and stroke-like episodes. Stroke-like episodes are cardinal features of the disease and are frequently accompanied by epilepsy, headache, myopathy, visual disturbances, hearing loss, diabetes, cardiomyopathy, gastrointestinal abnormalities, and cognitive impairment [77].

**Figure 6:**
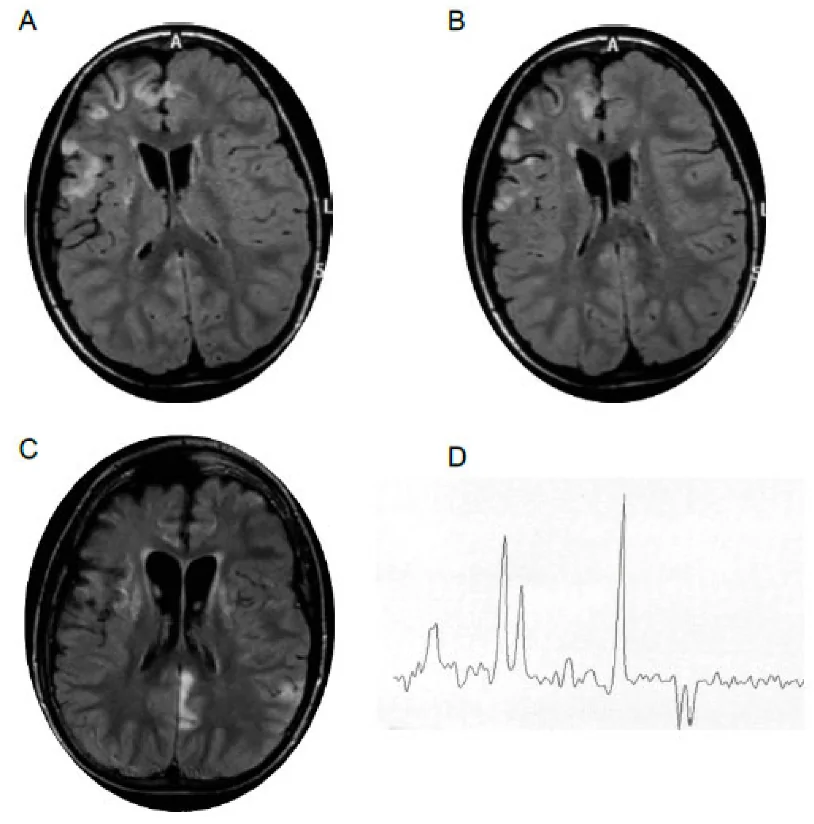
FLAIR imaging and MR spectroscopy in MELAS disease. (**A**,**B**) Axial FLAIR images demonstrating cortical and subcortical signal changes with a vascular pattern in the right frontal regions, along with involvement of the right putamen and left caudate head. (**C**) One-year follow-up axial FLAIR image showing interval development of focal atrophy in the previously described regions and the emergence of new signal abnormalities in the left parietal region. (**D**) MR spectroscopy with the voxel positioned over the affected area, demonstrating a lactate peak.

Brain MRI reveals multifocal ischemic lesions that do not necessarily follow vascular anatomy and are frequently associated with cortical atrophy. Acute cortical lesions in MELAS most commonly affect the primary visual, somatosensory, and auditory cortices, all regions of high neuronal density and metabolic demand [78]. Stroke-like lesions consistently demonstrate high signal intensity on DWI and variable ADC values (increased, normal, or decreased) [79] (Figure 6). Perfusion analysis (PWI and ASL) shows hyperperfusion in the acute phase and hypoperfusion in the chronic phase [80]. Spectroscopy analysis usually demonstrates reduced N-acetylaspartate signals and elevated lactate peaks [79].

## 17. Fabry Disease

Fabry disease is a multisystemic X-linked recessive disorder caused by deficiency of the enzyme alpha-galactosidase A, leading to the accumulation of glycosphingolipids, particularly within the endothelium and smooth muscle cells of vessels supplying the kidneys, lungs, myocardium, and central nervous system. Fabry disease is the second most frequent lysosomal storage disorder [81].

The disease usually begins during childhood or adolescence, although in some patients it may manifest only after the third decade of life. Early nonspecific manifestations include episodic pain in the extremities and a characteristic cutaneous lesion known as angiokeratoma corporis diffusum. These lesions are dark red to black, punctate papular lesions that predominantly involve the genital region, proximal thighs, and lower abdomen, while typically sparing the face, scalp, and palmoplantar regions [82].

Pain is a major symptom of the disease and may be described as chronic paresthesias or excruciating episodic pain beginning in the hands and feet. Hypohidrosis usually develops during the third decade of life and persists lifelong. Renal manifestations range from proteinuria to chronic kidney failure. Cardiovascular manifestations include arrhythmias, angina, and myocardial infarction [83]. Fabry crises may be precipitated by fever, exercise, fatigue, stress, and rapid temperature changes [84]. From a neurological perspective, the disease causes multiple ischemic strokes that are frequently interpreted as cryptogenic [85].

MRI typically demonstrates hyperintense lesions on T2-weighted and FLAIR sequences involving the periventricular white matter and centrum semiovale. Small vessel microangiopathy is the most common manifestation of cerebral vasculopathy in Fabry disease [86]. Silent lacunar infarcts are also frequently observed. Focal unilateral or bilateral hyperintensity of the pulvinar nuclei of the thalami on T1-weighted MRI is considered an almost pathognomonic sign of the disease [87]. Dilated arteriopathy of the vertebrobasilar system and cerebral microbleeds are common, although inconstant, neuroradiological features of the disease [88]. Definitive diagnosis is established through laboratory measurement of alpha-galactosidase A activity.

## 18. Migraine

Migraine may increase the risk of stroke by up to two-fold compared with the general population. This risk appears to be more pronounced in patients with poorly controlled disease and in those who experience more than 12 episodes per year [89,90]. Patients with migraine have up to a fourfold greater risk of developing white matter hyperintensities, which in turn are associated with an increased cumulative cerebrovascular risk [91].

In addition, patients with migraine frequently share other well-established stroke risk factors, including obesity, use of hormonal contraceptives, and the presence of a patent foramen ovale (PFO) [92,93,94].

A rare condition, often considered overestimated but nevertheless important to recognize, is migrainous infarction. This entity is defined by the presence of an ischemic cerebral lesion in patients with prolonged aura lasting more than 60 min and preferentially involves the posterior circulation. The pathophysiology remains incompletely understood, but it is believed that excessive release of calcitonin gene-related peptide (CGRP) may act on endothelial cells of cerebral vessels and on platelets, triggering the release of platelet-activating factor (PAF), a potent inflammatory mediator involved in platelet aggregation [95].

Migraine patients also have an increased risk of subclinical cerebellar infarcts, a risk that increases with attack frequency and is particularly evident in patients with migraine with aura (MA). Most of these infarcts are located within the border zones of the cerebellar arteries [96].

## 19. Malignancy

A wide variety of cancers may be associated with stroke in young individuals. The principal mechanisms include the hypercoagulable state induced by malignancy and the migration of tumor emboli into the cerebral circulation. The possibility of paradoxical embolism in patients with PFO and cancer is also recognized, given that venous thrombosis is more prevalent among oncologic patients. Another possible explanation, perhaps less relevant in younger populations, is the sharing of common risk factors for both diseases, such as smoking [97].

An American study demonstrated that approximately 1 in every 10 patients hospitalized for ischemic stroke had cancer as a comorbidity [98]. Another study revealed that nearly 20% of patients with cryptogenic stroke had an occult malignancy at the time of the cerebrovascular event [99]. The association is not always immediately evident, as stroke may precede the diagnosis of cancer. In some cases, the association with malignancy is established only years after the index stroke [100]. Neoplasms may also cause intracerebral hemorrhage [101]. The clinical presentation of cancer-related stroke is often indistinguishable from strokes caused by other etiologies.

Patients with cancer-related stroke usually present with multiple lesions involving multiple vascular territories [99]. Nevertheless, there are no highly specific radiological findings that definitively indicate a causal association between stroke and malignancy.

## 20. Infective Endocarditis

Stroke may be the first presentation of infective endocarditis and, evidently, represents the main cause of neurological complications in patients already diagnosed with the infection [102]. In the former scenario, maintaining a high degree of clinical suspicion during the acute phase is essential, since infective endocarditis constitutes an absolute contraindication to intravenous thrombolysis. Endocarditis caused by Staphylococcus aureus, the detection of incremental increases in vegetation size of at least 1 mm, high-intensity signals on transcranial Doppler, and the presence of comorbidities are recognized risk factors for cerebrovascular complications [103].

Infective endocarditis as a cause of stroke should be particularly suspected in patients presenting with acute focal neurological deficits associated with a history of fever, anemia, and abnormal cardiac auscultation, although not all these systemic signs and symptoms may necessarily be present [104].

On neuroimaging, one typically expects to identify multiterritorial septic emboli leading either to covert brain infarction or to overt stroke. Compared with stroke caused by atrial fibrillation, stroke related to infective endocarditis is characterized by smaller lesions, frequently exceeding 10 lesions in number, with predominant involvement of the bilateral anterior and posterior circulations [105]. In almost two-thirds of patients, the so-called “three-territory sign” may be observed, defined as an imaging pattern with ischemic lesions involving all three major cerebral vascular territories [106].

## 21. Cardiomyopathy and Cardiac Tumors

Within this category, several conditions have been associated with stroke in young individuals. Atrial myxoma, cardiac rhabdomyoma, atrial cardiomyopathy, aortic valve pathology, double-inlet ventricle, aortic coarctation, and left ventricular diverticulum have all been associated with stroke at a young age [107,108,109,110,111]. Atrial myxoma is a rare, benign primary heart tumor that can cause cardioembolic stroke due to fragments of the tumor breaking off and entering the brain [112]. Congenital heart disease in general has been linked to both ischemic and hemorrhagic stroke [113]. The clinical presentation of stroke secondary to structural cardiac disorders is often indistinguishable from stroke caused by other etiologies. Likewise, the radiological pattern is usually unable to define the specific etiology beyond identifying the typical “embolic pattern” of cerebral infarction.

## 22. Conclusions

Emerging neuroimaging techniques may further improve the diagnostic assessment and characterization of stroke in young adults. Among these, quantitative susceptibility mapping (QSM) provides quantitative information on magnetic susceptibility changes associated with tissue components such as deoxygenated hemoglobin and iron. In the setting of acute ischemic stroke, QSM-derived measures may also provide insights into regional oxygen extraction and help identify metabolically compromised tissue, potentially complementing conventional perfusion imaging in the assessment of the ischemic penumbra. These techniques may therefore offer additional diagnostic and pathophysiological information, particularly in complex or atypical cases of stroke in younger patients [114].

In conclusion, this review presents a summary of the main causes of stroke in young people, focusing primarily on their clinico-radiological diagnosis. These causes deserve attention because they are sometimes different from those of strokes in adults. They should be thoroughly investigated, as knowledge of the cause of stroke is the main pillar for effective secondary prevention. It is noteworthy that commonly used stroke etiological classification systems may be of limited utility in young adults. For example, under the TOAST classification, the vast majority of the 20 causes presented would be classified as “other determined etiology,” resulting in a single category that encompasses an extremely diverse and heterogeneous range of underlying conditions. This may substantially limit the ability of the classification to capture the distinct etiological spectrum of stroke in young adults.

Recently, the 2026 AHA/ASA Guideline for the Early Management of Patients With Acute Ischemic Stroke has dedicated a special session for the management of stroke in children, indicating the relevance of the topic and ratifying that prompt diagnosis is essential to provide the most personalized and evidence-based treatment in this population [111].

## Data Availability

No new data were created or analyzed in this study.
